# Cervical Spine Screening Based on Movement Strategies Improves Shoulder Physical Variables in Neck-Related Shoulder Pain Patients: A Secondary Analysis from an Observational Study

**DOI:** 10.3390/jcm14072433

**Published:** 2025-04-02

**Authors:** Alberto Roldán-Ruiz, Javier Bailón-Cerezo, Deborah Falla, María Torres-Lacomba

**Affiliations:** 1 Departamento de Fisioterapia, Facultad de Ciencias de la Salud, Universidad Francisco de Vitoria, Ctra. Pozuelo-Majadahonda Km 1800, Pozuelo de Alarcón, 28223 Madrid, Spain; 2 Physiotherapy and Nursing Department, Faculty of Medicine and Health Sciences, University of Alcalá, Alcalá de Henares, 28805 Madrid, Spain; maria.torres@uah.es; 3 Departamento de Fisioterapia, Centro Superior de Estudios Universitarios La Salle, Universidad Autónoma de Madrid, 28023 Madrid, Spain; javier.bailon@lasallecampus.es; 4 Physiotherapy in Women’s Health Research Group-FPSM, Physiotherapy Unit, University of Alcalá, Alcalá de Henares, 28805 Madrid, Spain; 5 Centre of Precision Rehabilitation for Spinal Pain (CPR Spine), School of Sport, Exercise and Rehabilitation Sciences, College of Life and Environmental Sciences, University of Birmingham, Birmingham B15 2TT, UK; d.falla@bham.ac.uk; 6 Ramón y Cajal Institute of Health Research-IRYCIS, University Hospital of Ramón y Cajal, 28034 Madrid, Spain

**Keywords:** shoulder pain, cervical spine, neck, range of motion, strength, function

## Abstract

**Background:** It is important to consider the cervical spine as a potential contributor to shoulder pain, indicating the paramount importance of screening the cervical spine in patients with shoulder pain. **Objectives:** To study the immediate effects of cervical spine screening (CSS) on the shoulder active range of motion, isometric strength and self-reported function in patients with neck-related shoulder pain. **Methods**: A secondary analysis was conducted on data from a previous study. A cervical contribution was considered if a ≥30% shoulder symptom modification of pain intensity (Numeric Pain Rating Scales) was recorded during the most painful shoulder movement after CSS. Pre–post measurements of the shoulder active range of motion (inclinometer) and shoulder isometric strength (dynamometer) were recorded in a single session. Self-reported shoulder function (Shoulder Pain and Disability Index) was assessed at a 1-week follow-up. **Results:** Among 60 participants, statistically significant changes were found for those with a cervical contribution (*n* = 30) for shoulder flexion and the abduction range of motion (*p* < 0.001), with a medium size effect (r = 0.55), and in internal rotation (*p* = 0.02) and external rotation at 0° abduction (*p* = 0.008), with a small size effect (r = 0.3 and 0.34, respectively). The self-reported shoulder function in those without a cervical contribution significantly declined from the pre to post measurements (*p* = 0.002), with a small size effect (r = 0.4). No statistically significant changes were found for the isometric strength in either group. **Conclusions**: In patients with shoulder pain classified as having a cervical contribution, CSS produces intrasession improvements in the active shoulder range of motion but not in the shoulder isometric strength or self-reported shoulder function.

## 1. Introduction

Shoulder pain constitutes the third most prevalent musculoskeletal pain condition [1] and is the leading cause of atraumatic upper limb complaints [2]. The shoulder pain incidence has been reported to be 14.7–29.3 in every 1000 patients in primary care [3,4]. The prevalence ranged from 41.2 to 48.4 in every 1000 people between the years 1998 and 2007, being higher in women than in men [4]. Up to 66.7% of people may develop shoulder pain at least once in their lifetime [5] and the symptoms often persist even beyond 18 months in 50% of those affected [6].

Although still scarce, the scientific research considering a cervical contribution in shoulder pain presentations is experiencing growing momentum [7,8,9,10]. Regional interdependence may explain the cervical contribution, as impairments in a body region distinct from the painful one may be related with a patient’s chief complaint [11,12]. Regarding the interaction between the neck and shoulder region, referred shoulder pain patterns have been observed from the cervical zygapophyseal joints [13,14,15,16], cervical intervertebral discs [17] and neck muscles [18].

Being guided by the anatomical region presenting with symptoms may therefore be misleading, as conditions of both the neck and shoulder region may overlap [19,20,21,22]. Moreover, shoulder orthopedic test results may turn into false positives in the presence of undiagnosed spinal disorders [23]. If pain of spinal origin is incorrectly interpreted, poor decision-making and inadequate patient management can result [24]. Similarly, directing treatment only over the shoulder in a patient with a cervical contribution might compromise the treatment effectiveness [8].

In that manner, from a clinical reasoning perspective, certain clinical findings may prompt a cervical spine evaluation to assess its potential contribution to shoulder pain. These include the presence of cervical pain prior the onset of shoulder symptoms [7], the coexistence of shoulder pain and cervical pain [16], a restricted cervical range of motion [25,26], symptom modification with cervical movements [25], the diagnosis of rotator-cuff-related shoulder pain [27], minimal or no reduction in shoulder range of motion [25,28], and a history of prior cervical surgery [29,30].

The effects of cervical treatment on shoulder symptoms have been examined, with the results showing that joint mobilization strategies such as postero-anterior or antero-posterior mobilizations, lateral glides and manipulations may enhance shoulder pain and disability [31,32,33,34,35,36,37], with different forms of manual therapy showing comparable effects [31]. Similarly, other cervical approaches, such as repeated movements [9,24,38,39] and spinal mobilization with arm movement [40], seem to be effective in managing shoulder symptoms. Despite the growing recognition of the potential role of the cervical spine in shoulder pain, and a recent Delphi study attempting to establish a consensus on spine screening in patients with shoulder pain—incorporating strategies such as assessing the changes in shoulder symptoms with neck movements or active cervical movements—[41], there is currently no standardized examination procedure to identify this clinical pattern [8].

The application of symptom modification strategies as both a diagnostic and a treatment-guiding approach has the potential to enhance clinical practice. By identifying specific structures or areas where treatment leads to symptom improvement, physical interventions can be more effectively directed [10]. Thus, the aim of this study was to investigate the immediate effects of cervical spine screening (CSS) based on movement strategies on the active shoulder range of motion, isometric shoulder strength, and self-reported shoulder function in patients with shoulder pain.

## 2. Methods

### 2.1. Study Design

This study constitutes a secondary analysis of a previous prospective observational study [7] approved by the Research Ethics Committee of Hospital Universitario Príncipe de Asturias, Spain (OE 25/2019) on 4 December 2019, conducted in compliance with the Declaration of Helsinki. The reporting adheres to the STROBE guidelines. Participants provided informed written consent prior to data collection.

### 2.2. Participants

Participants were recruited from two separate hospitals in Madrid and were consecutive eligible individuals seeking orthopedic/traumatology consultation for their shoulder pain. A total of 6 participants were recruited from Hospital Universitario Príncipe de Asturias and 54 from Clínica Cemtro, Madrid, Spain. From February 2020 to June 2022, participants were assessed by the same physiotherapist, who had six years of expertise in the assessment and conservative treatment of patients with shoulder pain.

Participants were required to be over 18 years old, suffering from shoulder pain during movement and scoring ≥3/10 on the Numeric Pain Rating Scale (NPRS) for their shoulder pain intensity.

They were excluded if they:

- had prior shoulder surgery, pain at rest that improved with movement, history of trauma, shoulder fractures, systemic diseases, radiculopathy, peripheral neuropathy, or radicular pain (including a score of >11 on the S-LANSS scale);
- had received local analgesic or anti-inflammatory injections in the previous six months or taken those medications on the examination day.

### 2.3. Variables

Baseline demographic and descriptive information was recorded, including age, sex, and the Spanish version of the Central Sensitization Inventory (CSI) [42], along with clinical characteristics such as prior and concomitant neck pain, symptoms extending to the upper limb, duration of symptoms, and whether their pain was unilateral or bilateral. The physical therapist also recorded several dependent variables during the clinical examination of the participants, as detailed below. The primary outcomes were the pain intensity, active shoulder range of motion and isometric shoulder strength. Self-reported shoulder function was considered a secondary outcome.

The shoulder pain intensity was assessed during the most painful shoulder movement, which was rated using a Numeric Pain Rating Scale (NPRS), an 11-point numerical scale with scores from 0 (no pain) to 10 (worst imaginable pain). The NPRS has adequate test–retest reliability (ICC = 0.63–0.92) and internal consistency (alpha coefficient = 0.84–0.98) [43,44]. A diagnosis of cervical contribution was considered if a ≥30% modification of the shoulder pain intensity was recorded after CSS; as in previous studies, a ≥30% threshold was employed to determine a significant change in shoulder symptoms [45,46].

**Figure 1:**
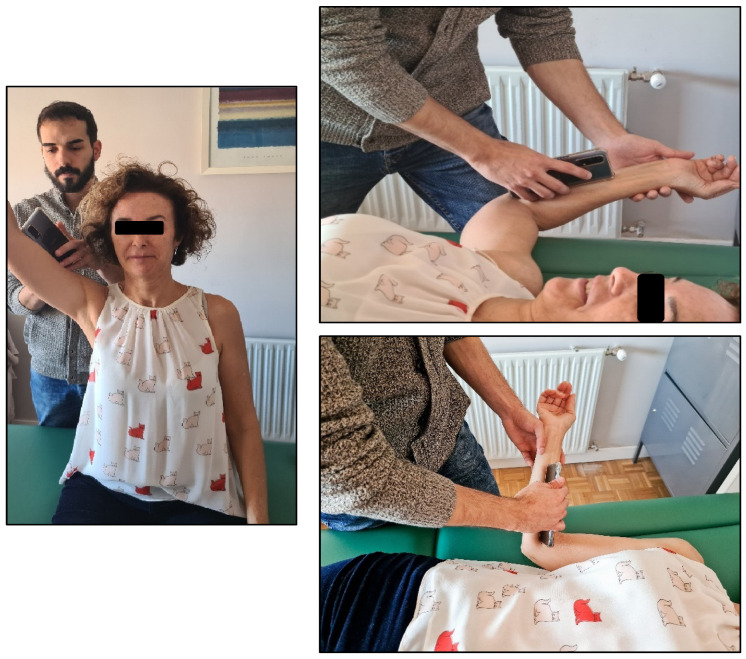
Shoulder external rotation in 90° abduction, external rotation in 0° abduction, and abduction measured with a smartphone inclinometer.

The shoulder active range of motion was assessed during the most painful shoulder movement using a smartphone inclinometer (www.plaincode.com, Plaincode Software Solutions, Gunzenhausen, Germany), where the use in shoulder flexion and abduction in a sitting position, external rotation at 0° abduction, external rotation at 90° abduction and internal rotation at 90° abduction in a supine position (Figure 1) has been validated, with results showing excellent inter-examiner reliability in symptomatic subjects (ICC > 0.80) [47].

**Figure 2:**
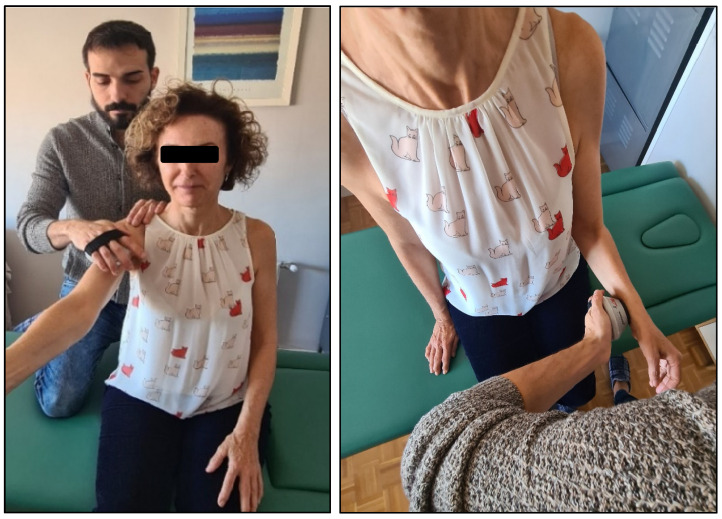
Shoulder flexion and internal rotation isometric strength measured with a dynamometer.

The isometric shoulder strength (Figure 2) was assessed using a hand-held dynamometer (MicroFET 2 MT Digital Handheld Dynamometer; Hoggan Health Industries, West Draper, UT, USA), which has been shown to have good to excellent intra-rater reliability for all shoulder strength tests (ICC = 0.87–0.99) [48,49]. As described by Roy et al., 2009 [50], the dynamometer was held over the anterior aspect of the humerus on the medial side for flexion and abduction, with the arm held at 90° and the participant in a seated position. For extension, the shoulder was at 0° of flexion–extension, and the dynamometer was placed over the posterior aspect of the humerus over the medial side. For external rotation, the dynamometer was held over the dorsal aspect of the forehand (or palmar aspect for internal rotation), close to the ulnar styloid process. Participants were asked to gradually increase their isometric force for 3 s, maintaining the contraction for a further 3 s and then relaxing. Two consecutive measurements with a 30 s rest period were carried out, with the mean force determined for each direction.

The self-reported shoulder function was assessed using the Shoulder Pain and Disability Index (SPADI), which is a questionnaire originally developed to measure shoulder pain and disability via 13 items [51]. It has been described as a questionnaire capable of adequately discriminating between patients with improving or worsening conditions [52]. The questionnaire was sent to the participants by email in Google Forms format prior to the CSS and after 1 week. A short-term follow-up was conducted for the self-reported shoulder function, given that substantial intrasession changes in this measure were not expected.

### 2.4. Procedure

The CCS consisted of different shoulder symptom modification strategies that were used with an evaluation approach to observe potential intrasession changes. After each intervention, the participant was asked to repeat the most painful movement and to report the pain intensity using the NPRS [43]. Immediately after completion of the CSS, the shoulder active range of motion and isometric strength were reassessed. The CSS was used to determine the presence of a cervical contribution, which was considered if a ≥30% modification of the shoulder pain intensity was recorded during the painful movement after the CSS. Based on this assessment, the included participants were then divided into the cervical contribution versus non-cervical contribution groups.

The CSS included the techniques presented in Table 1 (further detail is provided in Appendix A). All the strategies were applied to all the participants in the proposed order. If any strategy resulted in 100% symptom modification, the CSS was discontinued at that point.

### 2.5. Data Analysis

Statistical analyses were performed using SPSS version 27.0 (IBM Corp, Armonk, NY, USA). Continuous variables were presented as the mean ± standard deviation or the median with interquartile range, depending on their distribution, which was evaluated using the Kolmogorov–Smirnov (K-S) test. Categorical variables were summarized as the absolute and relative frequencies. Inferential analyses were conducted with a 95% confidence interval, considering a *p*-value < 0.05 as the threshold for statistical significance. The association between the pre- and post-screening values was analyzed using the Wilcoxon test for paired samples, due to the non-parametric distribution of these variables. The effect size was measured with the correlation coefficient r, calculated as r = Z/√n. According to Cohen, 1988 [53], an r value of 0.1 to 0.3 indicates a small effect size, 0.3 to 0.5 indicates a moderate effect size, and ≥0.5 indicates a large effect size.

As this was an exploratory secondary analysis, the sample size was not established a priori and the power estimations were limited by the non-parametric nature of the data.

## 3. Results

A total of 60 participants were assessed from 2020 to 2022 (mean age: 40.78 ± 13.45 years), 26 women and 34 men. The baseline clinical characteristics of the sample are shown in Table 2.

After the CSS, 30 out of the 60 participants experienced a ≥30% shoulder pain intensity modification, thus classifying 50% of the sample as having a cervical contribution.

### 3.1. Shoulder Range of Motion

When considering the whole sample and just those with a confirmed cervical contribution, the results revealed statistically significant changes after the CSS for all the shoulder range of motion variables except external rotation at 90° abduction (Table 3). The effect sizes were larger for those with a cervical contribution, especially for flexion and abduction (*p* < 0.001; r = 0.55).

Those without a cervical contribution did not show an improvement in the shoulder range of motion except for flexion, which was statistically significant (*p* = 0.007, r = 0.35). The effect sizes were small, except for flexion, which was moderate.

### 3.2. Shoulder Isometric Strength

The inferential analysis showed no statistically significant changes in the shoulder isometric strength immediately after the CSS in the whole sample or when people with or without a cervical contribution were considered separately. The effect sizes were small (Table 4).

### 3.3. Shoulder Function (SPADI)

The self-reported shoulder function showed no statistically significant improvement when considering the whole sample or those with or without a cervical contribution. However, those without a cervical contribution did show a statistically significant worsening of the SPADI score after 1 week (Table 5).

## 4. Discussion

To the best of our knowledge, this research is the first study analyzing the intrasession effects of CSS on shoulder physical variables in patients classified as having a cervical contribution to their shoulder pain. The results show how CSS based on movement strategies may enhance shoulder mobility but not shoulder strength or short-term self-reported shoulder function. This could be analyzed in relation to the results of the study by Cook et al., 2014 [54], the only study that, to the best knowledge of the authors’ knowledge, has shown the lack of value of cervical treatment in shoulder pathology, but without distinguishing between those with or without a cervical contribution to their shoulder pain condition.

### 4.1. Shoulder Range of Motion

Regarding the results of the active shoulder range of motion, it was observed that flexion, abduction, internal rotation, and external rotation at 0° of abduction obtained statistically significant changes when studying the whole sample and those with a confirmed cervical contribution. None of the similar studies analyzing cervical involvement in shoulder pain [9,10,24,38,39] have assessed the changes in the shoulder range of motion. Only Pheasant, 2016 [39], measured the baseline shoulder mobility in their case reports, but this was performed with a visual estimation and the post-intervention changes were not specifically described. However, improvements have been reported in the ROM, external rotation strength and abduction painful arc after cervical lateral glides [34,35], with this being a technique included in the current CSS.

It has been described that sustained or repeated cervical movements may modify shoulder symptoms, and if this is found to be a significant finding in the examination, cervical involvement can be considered a valid diagnosis [24,55]. In fact, given the difficulty of establishing a diagnosis based on the source or nociceptive structure responsible of the symptoms, this approach should be reserved for academic discussion, while in clinical practice, principles of reasoning based on the symptomatic response to mechanical stimuli during evaluation and treatment should be applied [55]. Nonetheless, the few studies considering the potential cervical spine involvement in shoulder pain have been based on examination and treatment under the premise of the McKenzie Mechanical Diagnosis and Therapy system [9,24,28,38,39], thus including only one part (repeated movements) of the CSS procedure that has been utilized in the current study.

### 4.2. Shoulder Isometric Strength

The shoulder isometric strength did not change following the CSS. There have been some reports of improvement in the shoulder strength and muscle activation after the implementation of treatment of the cervical spine, specifically with C5–C6 passive mobilization and C5–C6–C7 manipulation [32,33]. However, understandably, improvements in strength usually require a period of active exercise in order to induce neuromuscular adaptations to training, resulting in improved motor output. To assess the immediate changes, it may have been more relevant to assess the motor control, which is more likely to change immediately with an intervention [56].

The lack of change in the shoulder strength may be due to the assessment method. As manual therapy likely affects the pain-limited strength rather than the maximum contraction force, evaluating the pain-free contraction force, defined as the force generated up to the onset of pain, might have revealed different results.

### 4.3. Shoulder Function

The score on the SPADI questionnaire showed a statistically significant change for those without a cervical contribution to their shoulder pain, but the change [medians pre–post: 39.35 (31.21)–43.5 (33.57)] was below the minimal clinically important difference of this index, which is eight points [57]. In addition, the direction of change implied a worsening of the scores on the SPADI. However, measuring self-reported shoulder function within a single session may have limited value.

The absence of significant changes in the isometric strength or self-reported function raises questions about the clinical impact of CSS beyond mobility improvements. In this manner, the primary benefit of CSS may be restricted to an enhanced range of motion rather than broader functional improvements. Further research is needed to determine whether the intrasession changes derived from cervical screening may last in the long term. Similarly, longer-term interventions, rather than evaluations, might reveal additional benefits of addressing the cervical spine in shoulder physical variables in neck-related shoulder pain patients.

### 4.4. Strengths, Limitations and Future Research

This research used comprehensive CSS to classify whether or not people presenting with shoulder pain have a cervical contribution to their pain; previous studies have generally utilized repeated movements as the sole assessment strategy [9,24,38,39]. Considering the possibility of a cervical contribution in the symptomatic presentation of nontraumatic shoulder pain patients could enhance the overall management of this condition, since an approach based on the pathoanatomical model appears to be limited [56,58,59,60,61,62].

This is the first study to report the intrasession changes associated with a screening approach, rather than implementing treatment. However, the observed modifications in the physical variables could be influenced by unmeasured patient-specific factors, such as expectations, or placebo effects derived from the applied intervention. Future research should evaluate the role of these variables to determine whether the improvements reflect true physiological responses or expectancy-driven effects. Despite these limitations, the use of symptom modification strategies as a diagnostic, treatment, and reassessment method might be an option that aligns with clinical practice to guide treatment toward areas or structures that produce improvements [10]. Indeed, it has been reported that the intrasession changes in the pain intensity and range of motion may predict the future intersession changes in patients with neck disorders, and the same could happen with centralization phenomenon, i.e., distal symptoms diminish their expansion or disappear [63]. Likewise, it has been shown that intrasession changes higher than 36% are strongly associated with >50% changes in the Neck Disability Index after 96 h in patients with mechanical neck pain [64].

Nonetheless, this study has several noteworthy limitations. One significant concern is the representativeness of the sample, which may not adequately capture the diversity of a larger population as there was no sample size calculation. Additionally, the initial observational study that provided the dataset was not specifically designed to address the objectives of this research, leading to some inherent constraints in the data collection process. The reliance on secondary data also introduces challenges, such as the limited control over key variables and the possibility of missing or outdated information, so as not being able to control for confounding factors. These elements may impact the validity of the study’s conclusions, necessitating a cautious interpretation of the results. On the other hand, the short-term follow-up prevents us from drawing clear conclusions about whether the effects of cervical spine screening persist in the long term. Finally, it is necessary to mention that an unblinded assessor was not possible, as well as the assessor and treating therapist being the same person, which could lead to observer bias. To overcome these limitations, future research should focus on developing more robust and targeted study designs that address these challenges and enhance the reliability of the findings.

Future research observing the shoulder symptom changes when directing specific treatment to patients responding positively to CSS is needed. Similarly, studies with larger sample sizes with prospective designs and longer-term follow-ups are needed.

## 5. Conclusions

In people with shoulder pain with a cervical contribution, CSS produces intrasession improvements in the active shoulder range of motion but not in the shoulder isometric strength nor self-reported shoulder function. Future research with larger samples and longer follow-ups is required.

## Figures and Tables

**Table 1:** Proposed cervical spine screening.

Intervention	Included Variants
Postural change while performing painful movement	-
Repeated end-range movements	Cervical retraction, retraction plus extension and flexion
Spinal accessory mobilization with painful shoulder movement	Cervical global traction and lateral glide of C4 spinous process
Passive accessory mobilization	Posterior–anterior and anterior–posterior mobilizations and lateral glides
Neck flexor and extensor muscle activation	-

**Table 2:** Baseline sample clinical characteristics. Absolute frequency—*n* (%).

Variables	Descriptive Data
Clinical characteristics. Absolute frequency—*n* (%)	
Painful shoulder (left)	28 (46.7)
Upper limb referred pain	27 (45)
Unilateral pain	49 (81.7)
Previous neck pain	22 (36.7)
Concomitant neck pain	23 (38.3)
Symptoms duration	
<3 months	16 (26.7)
3–6 months	9 (15)
>6 months	35 (58.3)
Clinimetrics	
Central sensitization (CSI), >40 points	12 (20)
Shoulder function (SPADI)—median [IQR]	38.57 [28]
Pain intensity (NPRS)—median [IQR]	6 [2]

Abbreviatures: SD—standard deviation, IQR—interquartile range, CSI—Central Sensitization Inventory, SPADI—Shoulder Pain and Disability Index, NPRS—Numeric Pain Rating Scale.

**Table 3:** Pre–post changes in the shoulder active range of motion in the whole sample, those with a cervical contribution and those without a cervical contribution.

		V0 Median (IQR)	V1 Median (IQR)	*p*	Effect Size (r) *
Flexion	Whole sample (*n* = 60)	150.5 (37.25)	156.5 (34.25)	<0.001	0.46
	Cervical contribution (*n* = 30)	153.50 (30.50)	165 (27.75)	<0.001	0.55
	Non-cervical contribution (*n* = 30)	150 (42.75)	151 (46.25)	0.007	0.35
Abduction	Whole sample (*n* = 60)	155 (30)	160 (40.75)	<0.001	0.37
	Cervical contribution (*n* = 30)	155 (35.25)	170 (36.25)	<0.001	0.55
	Non-cervical contribution (*n* = 30)	155 (35)	155 (39.25)	0.833	0.03
External rotation at 0° abduction	Whole sample (*n* = 60)	62.43 (21.95) M (SD)	65.48 (20.29) M (SD)	0.010	0.23
	Cervical contribution (*n* = 30)	63.90 (18.76) M (SD)	69.43 (15.15) M (SD)	0.008	0.34
	Non-cervical contribution (*n* = 30)	61.06 (24.98) M (SD)	61.53 (23.99) M (SD)	0.461	0.10
External rotation at 90° abduction	Whole sample (*n* = 60)	84 (24.25)	85 (20.75)	0.363	0.08
	Cervical contribution (*n* = 30)	83 (24.25)	86 (19.25)	0.201	0.17
	Non-cervical contribution (*n* = 30)	84.5 (27.75)	83 (27.25)	0.843	0.03
Internal rotation at 90°	Whole sample (*n* = 60)	63.9 (18.73) M (SD)	66.95 (16.23) M (SD)	0.014	0.22
	Cervical contribution (*n* = 30)	64.97 (17.86) M (SD)	70.47 (13.63) M (SD)	0.020	0.30
	Non-cervical contribution (*n* = 30)	62.83 (19.81) M (SD)	63.43 (18.01) M (SD)	0.529	0.08

IQR—interquartile range; M—median; SD—standard deviation; V0—baseline mean values; V1—post-screening mean values; * r correlation coefficient [53].

**Table 4:** Pre–post changes in the shoulder isometric strength during the most painful movement in the whole sample, those with a cervical contribution and those without a cervical contribution.

	V0 Median (IQR)	V1 Median (IQR)	*p*	Effect Size (r) *
Whole sample (*n* = 60)	90 (77.87)	89 (61.75)	0.40	0.08
Cervical contribution (*n* = 30)	78.20 (44.93)	80.25 (46.25)	0.094	0.22
Non-cervical contribution (*n* = 30)	112 (67.75)	105 (66)	0.51	0.08

IQR—interquartile range; V0—baseline mean values; V1—post-screening mean values; * r correlation coefficient [53].

**Table 5:** Pre–post changes in the self-reported shoulder function in the whole sample, those with a cervical contribution and those without a cervical contribution.

	V0 Median (IQR)	V1 Median (IQR)	*p*	Effect Size (r) *
Whole sample (*n* = 60)	38.57 (27.95)	39 (33.77)	0.868	0.02
Cervical contribution (*n* = 30)	37.7 (29.97)	29.63 (33.01)	0.088	0.22
Non-cervical contribution (*n* = 30)	39.35 (31.21)	43.5 (33.57)	0.002	0.40

IQR—interquartile range; SPADI—shoulder pain and disability index; V0—baseline mean values; V1—post-screening mean values; * r correlation coefficient [53].

## Data Availability

The raw data supporting the conclusions of this article will be made available by the authors on request.

## Abbreviation

The following abbreviation is used in this manuscript:

CSS	Cervical spine screening

## Appendix A

### Appendix A.1. Cervical Spine Screening

After each strategy, patients were asked to repeat their most painful movement to reassess the pain intensity. After the completion of the whole CSS, the remaining dependent variables were also reevaluated.

1. Postural adjustment during painful movement: forward head posture and thoracic/lumbar slump were corrected. By maintaining this neutral cervical spine position, the participant performed the painful or restricted movement.
2. Repeated end-range movements: cervical retraction, retraction plus extension and flexion were performed 10 times, followed by reassessment of the painful or restricted movement. If the participant was unable to reach the end range, passive overpressure was applied by the examiner. Repeated end-range movements were conducted in all directions in the specified sequence unless a technique resulted in complete symptom resolution.
3. Spinal mobilization with shoulder movement:
  a. Cervical global traction: with the participant seated, the examiner positioned behind them applied gentle upward traction at the occiput using a bimanual grip. The participant then performed the painful or restricted movement while maintaining this mobilization.
  b. C4 lateral glide: with the participant seated, the examiner applied lateral pressure to the C4 spinous process in the direction opposite to the painful shoulder. This mobilization was maintained while the participant performed the painful or restricted movement.
4. Passive accessory mobilizations: techniques included posterior–anterior, anteroposterior, and lateral glides. The cervical segment selected for mobilization was the one that elicited symptom reproduction after three posterior–anterior mobilizations. If no symptoms were provoked, the segment associated with the greatest local discomfort was chosen [54]. Each mobilization was performed for 1 min.
5. Neck flexor (a) and extensor muscle (b) exercises while performing sets of contractions.
  a. Double chin movement (cranio-cervical flexion) was taught to the participant in a supine position. After this, he/she was placed standing with his/her back and head against a wall. A tennis ball was put between his/her head and the wall as feedback to better understand the exercise, and the subject was asked to perform the double chin movement while avoiding the ball falling. This was repeated 10 times.
  b. In a seated position, the subject was asked to place a band in his/her occiput and holding it forwards with his hands. Then, he/she was asked to perform a cervical retraction against the resistance of the band. This was repeated 10 times.
  c. As the CSS was an accumulation of strategies that could improve or worsen the subject’s baseline status, the possibility of stopping the CSS if the subject experienced an increase of ≥2 points in the NPRS score was contemplated.
